# Impact of cobalt and proline foliar application for alleviation of salinity stress in radish

**DOI:** 10.1186/s12870-024-04998-6

**Published:** 2024-04-16

**Authors:** Hira Inayat, Hassan Mehmood, Subhan Danish, Sulaiman Ali Alharbi, Mohammad Javed Ansari, Rahul Datta

**Affiliations:** 1 Department of Agronomy, Faculty of Agricultural Sciences and Technology, Bahauddin Zakariya University, Multan, Punjab Pakistan; 2 Department of Soil Science, Faculty of Agricultural Sciences and Technology, Bahauddin Zakariya University, Multan, Punjab Pakistan; 3 Department of Botany and Microbiology, College of Science, King Saud University, PO Box -2455, Riyadh, 11451 Saudi Arabia; 4 Department of Botany, Hindu College Moradabad (Mahatma Jyotiba Phule Rohilkhand University Bareilly), Moradabad, India; 5 Department of Geology and Pedology, Faculty of Forestry and Wood Technology, Mendel University in Brno, Zemedelska 1, Brno, 61300 Czech Republic

**Keywords:** Antioxidant enzymes, Cobalt sulfate, Proline, Salinity stress, Radish

## Abstract

Salinity stress ranks among the most prevalent stress globally, contributing to soil deterioration. Its negative impacts on crop productivity stem from mechanisms such as osmotic stress, ion toxicity, and oxidative stress, all of which impede plant growth and yield. The effect of cobalt with proline on mitigating salinity impact in radish plants is still unclear. That’s why the current study was conducted with aim to explore the impact of different levels of Co and proline on radish cultivated in salt affected soils. There were four levels of cobalt, i.e., (0, 10, 15 and 20 mg/L) applied as CoSO_4_ and two levels of proline (0 and 0.25 mM), which were applied as foliar. The treatments were applied in a complete randomized design (CRD) with three replications. Results showed that 20 CoSO_4_ with proline showed improvement in shoot length (∼ 20%), root length (∼ 23%), plant dry weight (∼ 19%), and plant fresh weight (∼ 41%) compared to control. The significant increase in chlorophyll, physiological and biochemical attributes of radish plants compared to the control confirms the efficacy of 20 CoSO_4_ in conjunction with 10 mg/L proline for mitigating salinity stress. In conclusion, application of cobalt with proline can help to alleviate salinity stress in radish plants. However, multiple location experiments with various levels of cobalt and proline still needs in-depth investigations to validate the current findings.

## Introduction

Salinity stress, caused by excessive salt in soil and water, hinders plant growth and reduces productivity [1, 2]. Higher levels of dissolved salts lead to degradation of agricultural land and decreased crop yield [3]. This stress affects plants by inhibiting water uptake and causing specific ion toxicity [4, 5], resulting in metabolic changes and decreased chloroplast activity [6–8]. Exacerbated by drought, global warming, and human activities, salinity poses a global challenge [9]. Addressing its impacts is crucial for food security and environmental sustainability [10]. Enhancing plant stress tolerance is essential for agriculture [11–13]. To combat salinity, cobalt sulfate supplemented with proline is gaining traction [14, 15].

Studies revealed that cobalt has dual role i.e., nutrients and stress producing metals [16–20]. Its application can increase water content in plants under salinity conditions. Its application can improve plant stress tolerance, enhancing growth and yield in agriculture and horticulture. Studies show that cobalt increases water content in plants under salinity conditions while decreasing photosynthesis and transpiration rates [16–18]. However, stomatal resistance increases. It might stimulate the synthesis or activation of antioxidant enzymes under salinity stress. These enzymes are crucial in neutralizing reactive oxygen species (ROS) and protecting cells from oxidative damage. It mitigates these adverse effects and maintains macro and micronutrient levels. Cobalt is essential for higher plants, synthesizing vitamin B12 for human and animal nutrition [21]. It does not accumulate in the human body with age, and its potential use in agriculture suggests avenues for addressing salinity hazards and improving crop productivity [22].

Plants have evolved defense mechanisms to survive in salt-stress environments [23–25]. They increase osmolytes like proline and ions to prevent water loss and toxicity [26–28]. Additionally, plants produce compatible osmolytes, low-molecular-weight organic molecules, in the cytosol and organelles, effectively functioning without disrupting intracellular biochemical processes. Proline, a crucial plant osmolyte, accumulates during stressful conditions such as salinity, oxidative stress, drought, and heavy metal exposure [29–32]. It helps plants tolerate stress by reducing reactive oxygen species and stabilizing membranes [33, 34], also acting as a regulatory molecule to trigger stress-alleviating responses [29, 30]. During salt stress, plants synthesize and accumulate proline to protect membranes, enzymes, and osmotic adjustment [35]. Foliar application of proline during seedling stages influences plant growth and physiological processes under salinity stress [36–40].

Radish (*Raphanus sativus* L.) is a valuable root vegetable crop belonging to the Brassicaceae family, widely grown worldwide due to its nutritional and medicinal benefits [41]. It is either an annual or biennial plant. This plant is known for its rich nutrition and therapeutic value [41]. Radishes are generally low in calories and rich in as calcium, magnesium, copper, manganese, potassium, vitamin B6, vitamin C, and folate. Its leaves and sprouts are commonly consumed as salads [42].

Considering the importance of the radish plant, this study investigates the effects of applying cobalt sulfate and proline as foliar on radish plant grown in salt affected soil. The aim of study is to assess cobalt and proline influence on radish growth, chlorophyll levels, antioxidant enzyme activity, and biochemical attributes when cultivated under salt stress conditions. It is hypothesized that combining cobalt with proline might mitigate salinity stress in radish. Current research is filling the knowledge gap regarding understanding the effectiveness of different concentrations of cobalt, individually and in combination with proline, as a foliar treatment to alleviate salinity stress.

## Materials and methods

### Experimental site

An experiment was done in 2023 at the ResearchSolution experimental site (30°09’41.6"N 71°36’38.0” E). The soil samples were collected from the research site for characterization. These samples were subjected to air-drying and passed through a 2-mm mesh to assess their physicochemical properties. Table 1 presents the physiochemical attributes of both the soil and irrigation water.

**Table 1:** Pre-experimental soil and irrigation characteristics

Soil	Values	References	Irrigation	Values	References
pH	8.29	[43]	pH	7.34	[44]
EC*e* (dS/m)	5.09	[45]	EC (µS/cm)	612	
SOM (%)	0.65	[46]	Carbonates (meq./L)	0.05	
TN (%)	0.025	[47]	Bicarbonates (meq./L)	7.66	
AP (µg/g)	8.34	[48]	Chloride (meq./L)	0.05	
EK (µg/g)	143	[49]	Ca + Mg (meq./L)	1.62	
ENa (µg/g)	231	[50]	Sodium (mg/L)	154	
Texture	Clay Loam	[51]			

AP = Available Phosphorus; TN = Total Nitrogen; ENa = Extractable Sodium; EK = Extractable Potassium; SOM = Soil Organic Matter

### Cobalt sulfate and Proline

The Cobalt (II) sulfate heptahydrate, specifically identified as Product Number C6768-2.5KG with Batch Number 0000300865, was acquired from a certified Sigma dealer in Multan. This product originates from Source Batch 0000266495 and was associated with a CAS Number of 10026-24-1 and an MDL Number of MFCD00149657. The proline was identified as L-Proline ReagentPlus, Product Number: P0380-100G, Batch Number: 0000321643, Source Batch: SLCR1010, CAS Number: 147-85-3 and MDL Number: MFCD00064318.

### Treatment plan and experimental design

There were two proline levels, i.e., No proline and with proline (0.25 mM). Four treatments, i.e., control, 10 mg/L CoSO_4_, 15 mg/L CoSO_4_ and 20 mg/L CoSO_4_, were applied in 4 replicates following a completely randomized design (CRD).

### Seed collection and sterilization

The radish seeds utilized in the study were purchased from a local seed supplier. The seeds were sterilized with a 5% sodium hypochlorite solution, followed by three washes using 95% ethanol. Subsequently, the seeds underwent three rinses in deionized water to remove residual sterilizing agentsClick or tap here to enter text.

### Seeds sowing and thinning

A total of 4 seeds were sown on 15 February 2023, with each pot containing 12 kg of soil. After germination, the number of seedlings in each pot was reduced to 2 through thinning.

### Fertilizer

During sowing, it is advisable to incorporate well-decomposed cow dung into the soil along with specific amounts of nutrients per acre. This includes nitrogen at a rate of 25 kg (0.37 g/12 kg soil) using urea and phosphorus at 12 kg (0.18 g/12 kg soil) using single superphosphate.

### Irrigation

The trial aimed to replicate normal soil moisture conditions (65% Field Capacity) using a moisture meter (YIERYI 4 in 1; Shenzhen, Guangdong Province, China), following a methodology recommended by the study [52].

### Harvesting and data Collection

After 60 days of sowing, plants were harvested for data collection. The fresh weights of both shoots and roots were promptly measured post-harvest. Subsequently, samples were subjected to oven-drying at 65 °C for 72 h to ensure consistent weight for determining the dry mass of both the shoot and root components.

### Chlorophyll contents

We measured chlorophyll levels in fresh plant leaves using the Arnon method [53]. We used 80% acetone to extract chlorophyll and then measured absorbance at 663 and 645 nm wavelengths. Specific formulas were used to calculate the amounts of chlorophyll a, chlorophyll b, and total chlorophyll.

Chlorophyll a (mg/g) = ((12.7 × A663) – (2.69 × A645) ×V)/ (1000 ×W)

Chlorophyll b (mg/g) = ((22.9 × A645) – (4.68 × A663) ×V)/ (1000 ×W)

Total Chlorophyll (mg/g) = 20.2(OD 645) + 8.02(OD 663) ×V/1000 (W)

### Antioxidants

SOD activity was measured by observing the reduction of nitro blue tetrazolium (NBT) at 560 nm [54]. POD activity was determined by following the method outlined by [55] and measuring absorbance at 420 nm. CAT activity was assessed by observing the decrease in absorbance at 240 nm during the breakdown of H_2_O_2_ [56]. APX activity was determined by monitoring ascorbate oxidation in the presence of H_2_O_2_ at 290 nm [57]. MDA levels were evaluated by mixing the sample extract with thiobarbituric acid (TBA) to create a colored complex. The absorbance of this complex was measured at 532 nm to determine MDA content [58]. We measured the proline content in plant tissue using a colorimetric assay. This involved reacting proline with ninhydrin, following a method outlined by [59]. The activity of GR can be measured spectrophotometrically by monitoring the reduction of oxidized glutathione (GSSG) to reduced glutathione (GSH) using NADPH as a cofactor [60, 61]. The content of GSH can be determined using spectrophotometric methods based on its reaction with 5,5’-dithiobis-(2-nitrobenzoic acid) (DTNB), as described by [60]. The content of ASA can be measured spectrophotometrically based on its oxidation to dehydroascorbic acid (DHA) using 2,6-dichlorophenolindophenol (DCPIP), as described by [62].

### Electrolyte Leakage

We rinsed the leaves with deionized water to conduct electrolyte leakage analysis to remove any surface impurities. We then obtained uniform leaf segments weighing approximately one gram each using a steel cylinder with a 1 cm diameter. These leaf segments were placed individually in test tubes containing 20 ml of deionized water. The test tubes were maintained at 25 °C for 24 h to allow electrolytes to diffuse from the leaf tissues into the surrounding water. After the incubation period, we measured the water solution’s electrical conductivity (EC1) using a pre-calibrated EC meter. Next, the test tubes were heated in a water bath at 120 °C for 20 min, and the second electrical conductivity (EC2) was determined [63].

$$Electrolyte{\rm{ }}\ Leakage{\rm{ }}\left( \% \right){\rm{ }} = {\rm{ }}\left( {EC1/EC2} \right){\rm{ }} \times {\rm{ }}100$$

### Relative water content

Relative water content (RWC) in plant tissue is determined by comparing the water content of a sample with its fully hydrated weight and its turgid weight after water immersion [64, 65]. The formula for calculating RWC is:

$$RWC{\rm{ }} = {\rm{ }}\left( {TW - DW} \right)/\left( {FW - DW} \right){\rm{ }} \times {\rm{ }}100$$

### Statistical analysis

The linear mixed model was used. The cobalt and proline were considered fixed effects, and replication was considered random. The analysis was performed in Origin software [66]. Means were compared using Tukey’s multiple comparison tests at a significance level of *p* ≤ 0.05. The statistical analysis was conducted using OriginPro 2021 [67]. Paired comparisons and cluster plots were also made using OriginPro 2021.

## Results

### Shoot and root length, plant fresh and dry weight

In no proline, 10 CoSO_4_ (∼ 7%, ∼ 14%, ∼ 16%, and ∼ 38%) and 15 CoSO_4_ (∼ 14%, ∼ 27%, ∼ 12%, and ∼ 69%) showed an increase in shoot and root length, plant fresh and dry weight, over control respectively. The 20 CoSO_4_ (∼ 23%, ∼ 42%, ∼ 22%, and ∼ 101%) showed maximum increase in shoot and root length, plant fresh and dry weight, respectively, compared to control. Under proline, a significant enhancement of ∼ 8%, ∼ 9%, ∼ 5%, and ∼ 15% in 10 CoSO_4_, 14%, ∼ 16%, ∼ 13%, and ∼ 24% in 15 CoSO_4_ and ∼ 20%, ∼ 23%, ∼ 19%, and ∼ 41% in 20CoSO4 from control in shoot and root length, plant fresh and dry weight, respectively (Table 2).

**Table 2:** Impacts of different levels of cobalt sulphate (10, 15, 20 mg/L) without and with proline on shoot and root length, plant fresh and dry weight, protein, and carbonyl content attributes of radish cultivated in salinity stress. Values are means of 3 replicates ± standard deviation (SD).

Applications of cobalt sulphate	Shoot length (cm)		Root length (cm)		Plant fresh weight (g)	
	No Proline	Proline	No Proline	Proline	No Proline	Proline
Control	14.82 ± 0.33a	19.62 ± 0.45a	6.79 ± 0.35a	10.54 ± 0.33a	9.49 ± 0.28a	12.48 ± 0.30a
10CoSO_4_	15.83 ± 0.45b	21.15 ± 0.45b	7.76 ± 0.19b	11.48 ± 0.40b	10.08 ± 0.16b	13.14 ± 0.23b
15CoSO_4_	16.92 ± 0.48c	22.45 ± 0.55c	8.62 ± 0.26c	12.18 ± 0.15c	10.66 ± 0.27c	14.12 ± 0.26c
20CoSO_4_	18.28 ± 0.41d	23.49 ± 0.43d	9.65 ± 0.23d	12.98 ± 0.33d	11.54 ± 0.25d	14.85 ± 0.28d

Applications of cobalt sulphate	**Plant dry weight (g)**		**Protein content (mg/g FW)**		**Carbonyl content (µmol/g FW)**	
	**No Proline**	**Proline**	**No Proline**	**Proline**	**No Proline**	**Proline**
Control	1.33 ± 0.20a	3.12 ± 0.14a	4.34 ± 0.78a	12.25 ± 0.74a	0.41 ± 0.04a	0.8 ± 0.04a
10CoSO_4_	1.84 ± 0.06b	3.57 ± 0.09b	6.02 ± 0.52b	14.02 ± 0.45b	0.54 ± 0.04b	0.89 ± 0.04b
15CoSO_4_	2.26 ± 0.12c	3.87 ± 0.06c	7.46 ± 0.52c	15.25 ± 0.50c	0.62 ± 0.03c	0.98 ± 0.01c
20CoSO_4_	2.68 ± 0.20d	4.39 ± 0.06d	9.85 ± 0.93d	17.26 ± 0.84d	0.72 ± 0.03d	1.08 ± 0.06d

### Chlorophyll a, b, total chlorophyll, and carotenoid

**Fig. 1:**
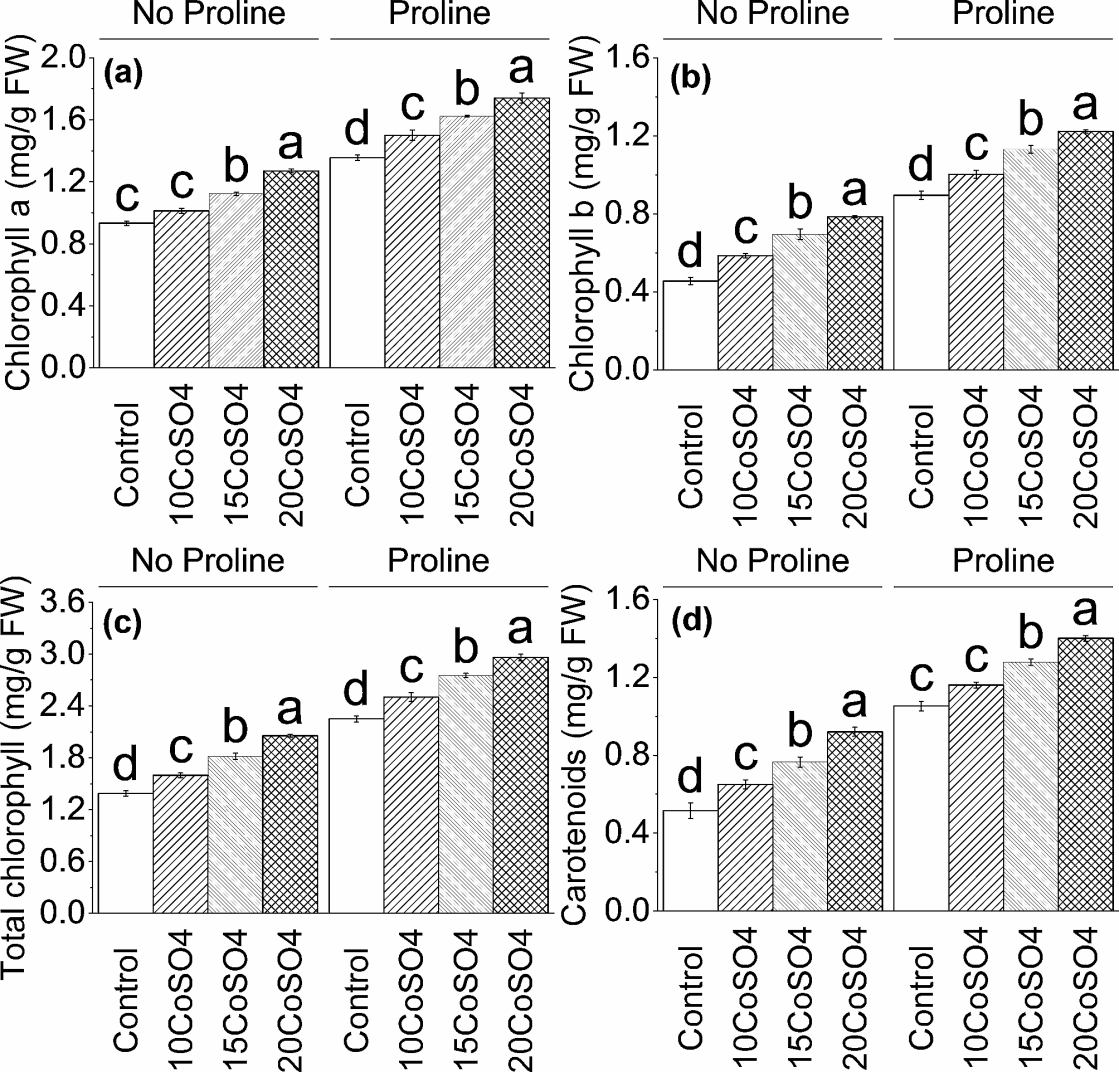
Effect of different levels of CoSO_4_ (10, 15 and 20 mg/L) on chlorophyll a (**a**), Chlorophyll b (**b**), Total chlorophyll (**c**) and Carotenoids (**d**) of radish plant with and without the application of proline. Bars are means of 4 replicates ± SE. Difference letters on bars showed significant changes at *p* ≤ 0.05: *Tukey’s test*. CoSO_4_: Cobalt Sulphate

Applying 10 CoSO_4_ showed ∼ 9%, ∼ 29%, ∼ 15%, and ∼ 26%, 15 CoSO_4_ caused ∼ 20%, ∼ 53%, ∼ 31%, and ∼ 49%, while 20 CoSO_4_ resulted ∼ 36%, ∼ 73%, ∼ 48%, and ∼ 79% enhancement compared to control in chlorophyll a, chlorophyll b, total chlorophyll, and carotenoid respectively without proline. A significant enhancement was observed under proline in chlorophyll a, chlorophyll b, total chlorophyll, and carotenoid where 10 CoSO_4_ ∼ 11%, ∼ 12%, ∼ 11%, and ∼ 10%, 15 CoSO_4_ ∼ 20%, ∼ 26%, ∼ 22%, and ∼ 21% and 20CoSO_4_ ∼ 28%, ∼ 37%, ∼ 32%, and ∼ 33% were applied over control respectively (Fig. 1).

### Relative water content, Electrolyte leakage, H_2_O_2_ and MDA

**Fig. 2:**
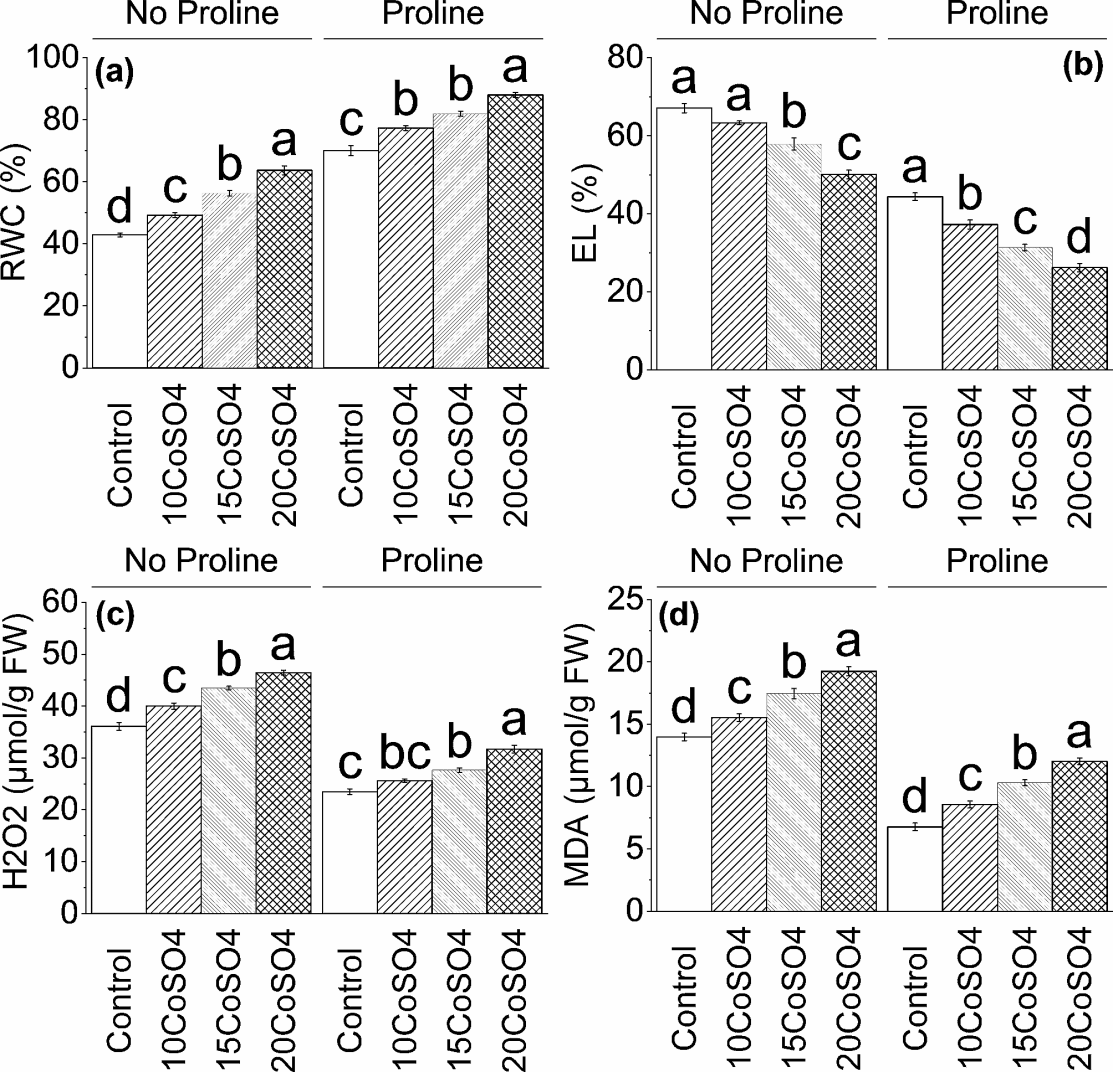
Effect of different levels of CoSO_4_ (10, 15 and 20 mg/L) on Relative water content (**a**), Electrolyte leakage (**b**), Hydrogen per oxide (**c**) and MDA (**d**) of radish plant with and without the application of proline. Bars are means of 4 replicates ± SE. Difference letters on bars showed significant changes at *p* ≤ 0.05: *Tukey’s test*. CoSO_4_: Cobalt Sulphate, H_2_O_2_: Hydrogen Peroxide, MDA: Malondialdehyde

Without proline, 10 CoSO_4_ treatment resulted in ∼ 15% enhancement in relative water content, respectively, than control. Treatment 15 CoSO_4_ showed ∼ 13%, and 20 CoSO_4_ caused a ∼ 49% increase over control in relative water content, respectively. Furthermore, with proline, 10 CoSO_4_ showed ∼ 10% while 15 CoSO_4_ caused ∼ 17% while 20 CoSO_4_ acid resulted in ∼ 26% increase in relative water content, respectively, compared to the control (Fig. 2).

Without proline, 10 CoSO_4_ treatment resulted in ∼ 6% reduction in electrolyte leakage, respectively, than control. Treatment 15 CoSO_4_ showed ∼ 14%, and 20 CoSO_4_ caused a ∼ 25% decrease over control in electrolyte leakage, respectively. Furthermore, under proline, 10 CoSO_4_ showed ∼ 16%, 15 CoSO_4_ caused ∼ 29%, while 20 CoSO_4_ acid resulted in ∼ 41% decrease in electrolyte leakage, respectively, compared to the control (Fig. 2).

In the presence of proline, 10 CoSO_4_ treatments they resulted in ∼ 11% and ∼ 11% enhancement in H_2_O_2_ and MDA, respectively, than control. Treatment 15 CoSO_4_ showed ∼ 21% and ∼ 25%, and 20 CoSO_4_ caused ∼ 29% and ∼ 38% increase over control in H_2_O_2_ and MDA, respectively. Furthermore, with proline, 10 CoSO_4_ showed ∼ 9% and ∼ 27%, 15 CoSO_4_ caused ∼ 18% and ∼ 52%, while 20 CoSO_4_ acid resulted in ∼ 35% and ∼ 78% increase in H_2_O_2_ and MDA, respectively, compared to control (Fig. 2).

### SOD, POD, CAT, and APX

**Fig. 3:**
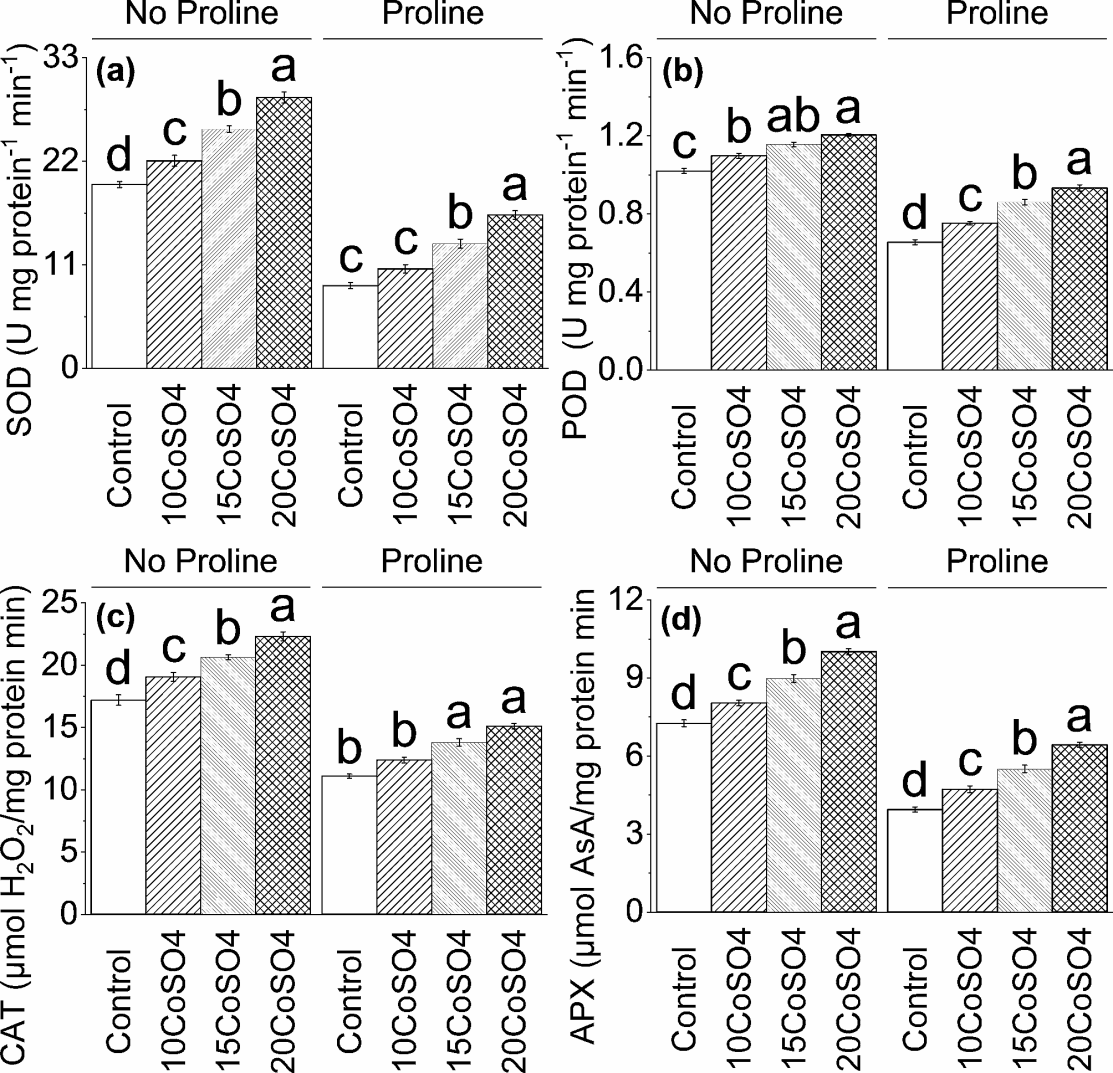
Effect of different levels of CoSO_4_ (10, 15 and 20 mg/L) on SOD (**a**), POD (**b**), APX (**c**), and CAT (**d**) of radish plant with and without the application of proline. Bars are means of 4 replicates ± SE. Difference letters on bars showed significant changes at *p* ≤ 0.05: *Tukey’s test*. CoSO_4_: Cobalt Sulphate, POD: Peroxidase, SOD: Superoxide Dismutase, APX: Ascorbate Peroxidase, CAT: Catalase

A significant increase was noted in SOD, POD, CAT, and APX when 10 CoSO_4_ ∼ 13%, ∼ 8%, ∼ 11%, and ∼ 11%, 15 CoSO_4_ ∼ 30%, ∼ 13%, ∼ 20%, and ∼ 24% and 20 CoSO_4_ ∼ 47%, ∼ 18%, ∼ 30% and ∼ 38% were applied to control respectively under no proline. In the case of Proline, SOD, POD, CAT, and APX showed an enhancement of ∼ 20%, ∼ 15%, ∼ 12%, and ∼ 20% in 10 CoSO_4_ ∼ 51%, ∼ 31%, ∼ 24%, and ∼ 40% in 15 CoSO_4_ and ∼ 87%, ∼ 42%, ∼ 36% and ∼ 63% in 20CoSO_4_ over control (Fig. 3).

### GR, proline, GSH and AsA

**Fig. 4:**
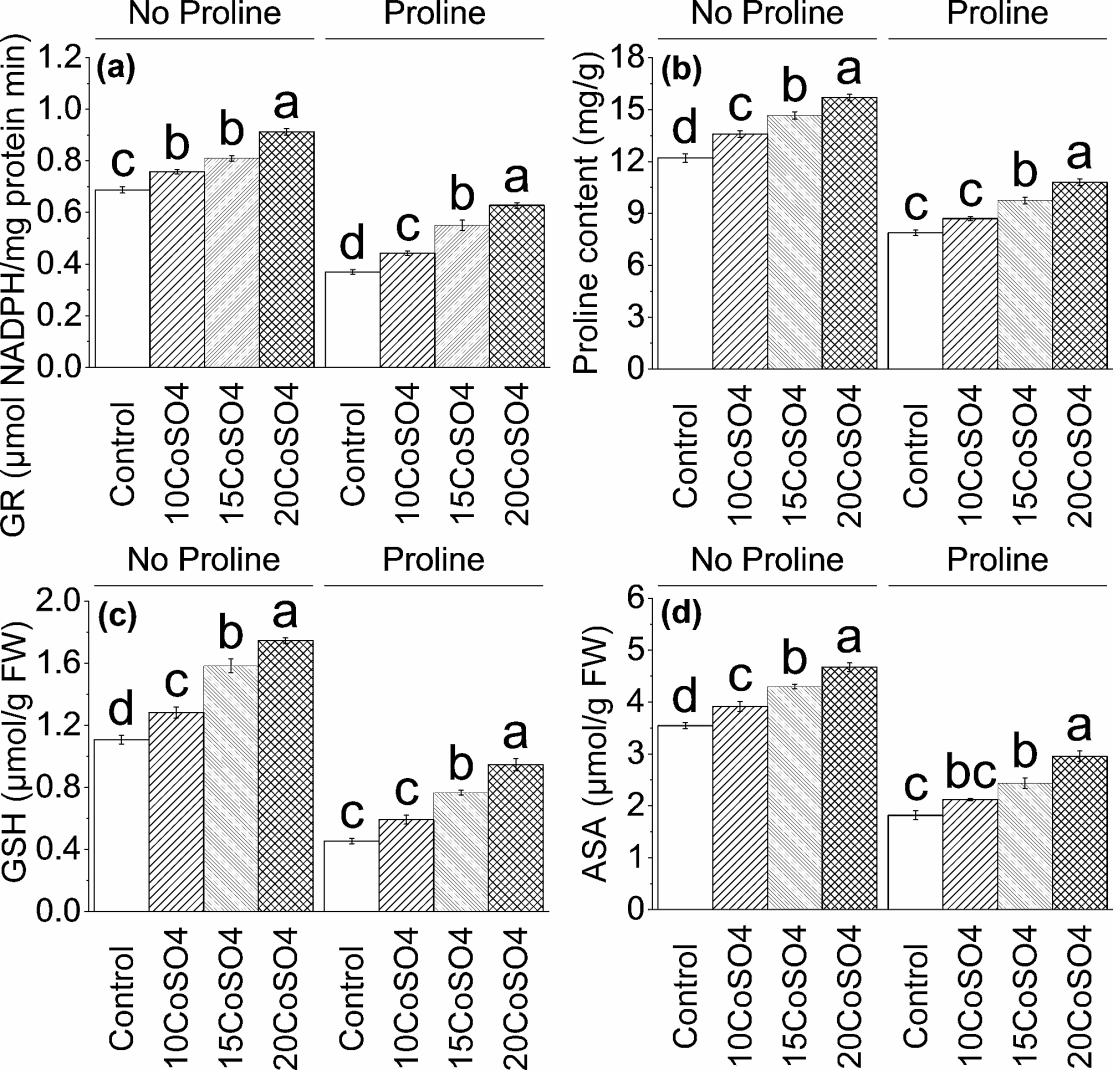
Effect of different levels of CoSO_4_ (10, 15 and 20 mg/L) on GR (**a**), proline (**b**), GSH (**c**), and ASA (**d**) of radish plant with and without the application of proline. Bars are means of 4 replicates ± SE. Difference letters on bars showed significant changes at *p* ≤ 0.05: *Tukey’s test*. CoSO4: Cobalt Sulphate, GR: Glutathione Reductase, GSH: Reduced Glutathione, ASA: Ascorbic Acid

For GR, proline, GSH, and AsA concentration at no proline, an increase was noted in 10 CoSO_4_ (∼ 10%, ∼ 11%, ∼ 16%, and ∼ 11%), 15 CoSO_4_ (∼ 18%, ∼ 20%, ∼ 43%, and ∼ 21%) and 20 CoSO_4_ (∼ 33%, ∼ 29%, ∼ 58%, and ∼ 32%) than control respectively. On the other hand, at proline, an enhancement of ∼ 20%, ∼ 10%, ∼ 30%, and ∼ 17% in 10 CoSO_4_, ∼ 49%, ∼ 24%, ∼ 69%, and ∼ 34% in 15 CoSO_4_ and ∼ 70%, ∼ 37%, ∼ 108%, and ∼ 62% in 20 CoSO_4_ was noted in GR, proline, GSH and AsA concentration over control (Fig. 4).

### Protein and carbonyl content

Results showed that protein and carbonyl content was significantly improved where 10 CoSO_4_ (∼ 39% and ∼ 33%), 15 CoSO_4_ (∼ 72%, and ∼ 54%) and 20 CoSO_4_ (∼ 127%, and ∼ 78%), respectively, over control under no proline. At proline, applying 10 CoSO_4_ (∼ 14% and ∼ 11%), 15CoSO_4_ (∼ 24% and ∼ 23%), and 20CoSO_4_ (∼ 41% and ∼ 35%) caused significant enhancement in protein and carbonyl content over control, respectively (Table 2).

### Convex Hull

**Fig. 5:**
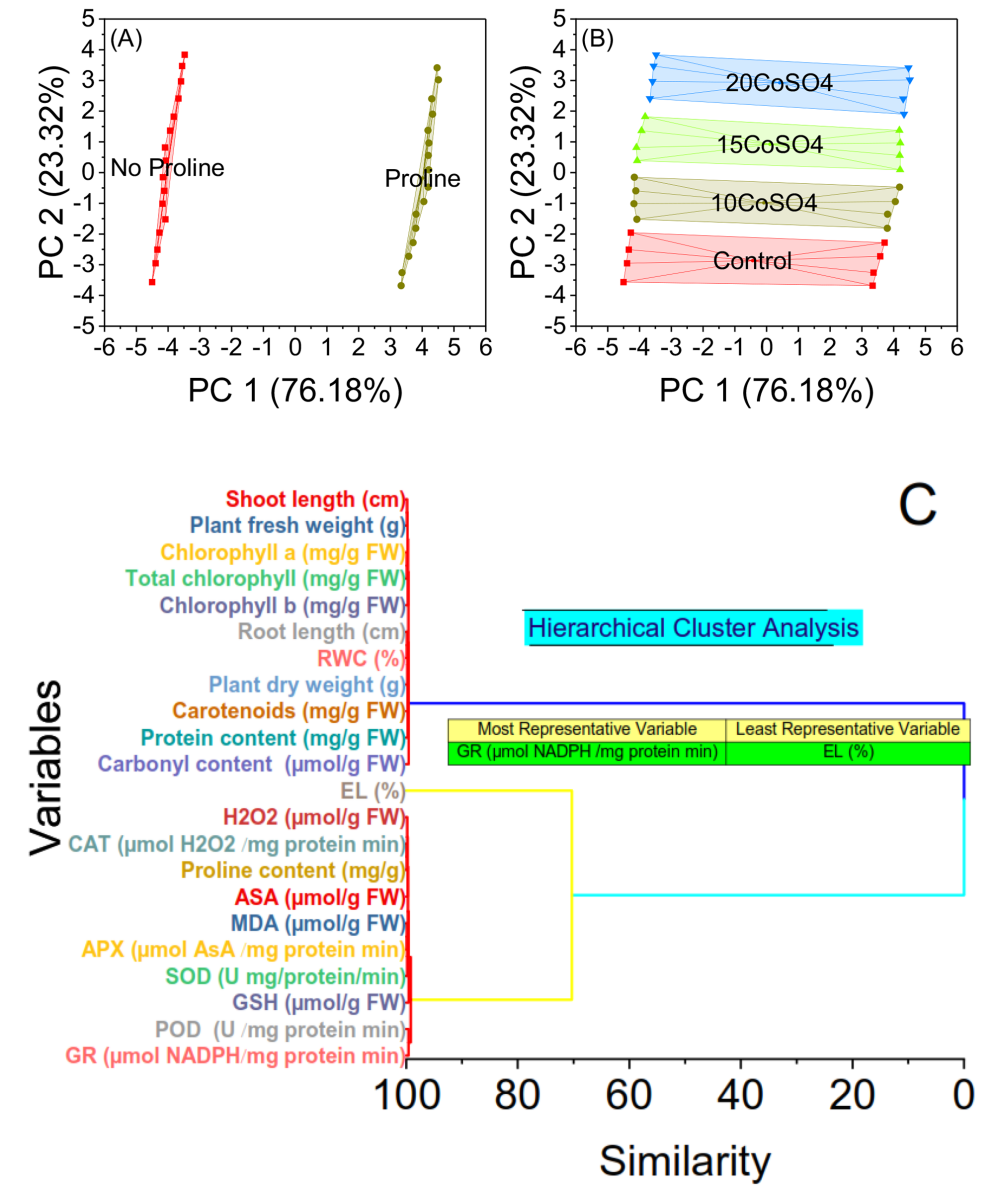
Convex hull cluster plots for treatments (**A**), proline (**B**), and hierarchical cluster plots for the studied attributes (**C**)

Samples labelled as no proline exhibit negative scores on both PC1 and PC2, indicating lower proline content, while samples labelled proline display positive scores on both components, suggesting higher proline content. This separation explains that proline content contributes to the observed variability among samples. Specifically, the PC1 axis, which accounts for 76.18% of the total variance, appears to reflect differences related primarily to proline content. The PC2 axis, responsible for 23.32% of the variance, represents additional variability beyond proline content (Fig. 5A). In the plot, it is observed that the control group is clustered separately from the groups treated with different concentrations of cobalt sulfate (CoSO_4_). Specifically, the control group is characterized by negative scores along both PC1 and PC2 axes, indicating lower levels of the analyzed parameters. In contrast, treatments with CoSO_4_, particularly at higher concentrations, show positive scores along both axes, suggesting elevated levels of the measured parameters. Among the CoSO_4_ treatments, there appears to be a trend of increasing scores with increasing concentration of CoSO_4_, indicating a dose-dependent response (Fig. 5B).

### Hierarchical cluster plot

The analysis indicates varying levels of similarity among physiological parameters. CAT activity and proline content show a low similarity (0.09517), while MDA concentration and APX activity exhibit slightly higher similarity (0.11681). Chlorophyll a and total chlorophyll closely resemble each other (0.1182); shoot length, plant fresh weight, and root length/relative water content share similarities (0.15281 and 0.16208). Superoxide dismutase activity and hydrogen peroxide concentration show similarities of 0.1736 and 0.18088, respectively. Plant dry weight/carotenoid content have relatively higher similarities (0.20733 and 0.22728). Ascorbate concentration/protein content share higher similarity (0.2494 and 0.25318), with chlorophyll b notably similar (0.3156). Peroxidase (POD) activity/glutathione reductase activity exhibits relatively high similarity (0.41363), while carbonyl content/glutathione concentration shares notable similarity (0.42957 and 0.44247). Electrolyte leakage demonstrates exceptionally high similarity (29.69999). Treatments 41 and 43 reveal extraordinarily high similarity coefficients of 99.57043 and 70.30001, suggesting potential correlations between them (Fig. 5C).

## Discussion

### Salinity stress

Salinity, drought, and global warming pose significant challenges to agricultural productivity worldwide [15]. Among these environmental stressors, soil salinity is a prominent issue affecting crop growth, production, and yield. Addressing salinity is imperative for ensuring food security in the face of these challenges [68, 69]. The effects of salinity stress on plants encompass various alterations in morphological parameters and biochemical processes. These alterations include reductions in root and shoot length, vegetable production, chlorophyll content, and changes in secondary metabolites such as oxidative compounds, signal molecules, and hormones [70, 71]. Salinity adversely affects the germination rate, germination percentage, and growth of seedlings.

Moreover, salinity induces oxidative stress within plants, disrupting plant metabolism, reducing soluble sugar content, and decreasing chlorophyll content [72]. Mechanistically, the impact of salinity stress on radish plants involves several interconnected pathways and physiological responses [73, 74]. Firstly, salinity stress disrupts the osmotic balance within plant cells, leading to water loss and impaired nutrient uptake [75]. This disruption triggers oxidative stress, where the excessive accumulation of reactive oxygen species (ROS) damages cellular components, including proteins, lipids, and DNA. Additionally, salinity stress alters hormonal balances, such as abscisic acid (ABA), which regulates plant responses to environmental stresses [73].

Furthermore, salinity stress influences the expression of genes involved in ion homeostasis, osmotic regulation, and stress responses [76]. For instance, the upregulation of genes encoding ion transporters facilitates the removal of toxic ions from cells, while the activation of stress-responsive genes helps plants cope with adverse conditions. In response to salinity stress, plants accumulate compatible solutes, such as proline and glycine betaine, to maintain cellular osmotic balance and protect against dehydration [77]. Moreover, the modulation of antioxidant defense systems, including enzymes such as catalase, superoxide dismutase, and peroxidase, is crucial for scavenging ROS and mitigating oxidative damage under salinity stress conditions. These mechanisms collectively enable radish plants to adapt to and mitigate the detrimental effects of salinity stress, although with varying degrees of success depending on the plant’s genetic makeup and environmental conditions. In the control treatment, similar results were observed, wherein salinity stress significantly decreased the growth attributes, chlorophyll contents, relative water content and various physiological and biochemical processes in the roots and shoots of radish.

### Cobalt sulfate

In our investigation of the application of cobalt foliar for alleviating salinity stress in Radish plants, we observed significant positive effects across various parameters. Cobalt sulfate application notably enhanced shoot length, root length, plant fresh weight, and plant dry weight in radish plants under salt stress conditions. This enhancement suggests that cobalt is pivotal in promoting overall plant growth and development, even in salinity stress [18, 78]. Mechanistically, cobalt likely influences cell division and elongation processes, thereby contributing to increased shoot and root length and enhanced plant fresh and dry weight [79, 80]. Additionally, Cobalt-treated radish plants exhibited improved chlorophyll a, chlorophyll b, total chlorophyll, and carotenoid contents, indicating enhanced photosynthetic efficiency and stress tolerance. Cobalt may enhance chlorophyll synthesis and protect chlorophyll molecules from degradation, thereby maintaining optimal photosynthetic activity under stressful conditions [78, 81].

Moreover, cobalt foliar treatment positively influenced relative water content (RWC) and reduced electrolyte leakage (EL), indicative of improved water status and membrane integrity [82]. Furthermore, Cobalt application increased levels of hydrogen peroxide (H_2_O_2_) and malondialdehyde (MDA), markers of oxidative stress-induced damage. Cobalt likely enhances the activity of antioxidant enzymes and scavenges reactive oxygen species, thereby protecting plant cells from oxidative damage and maintaining cellular integrity [83]. In the case of Biochemical parameters, the mechanism of action for GSH, ASA, GR, and similar compounds involves their functions in regulating oxidative stress and cellular balance. GSH acts as an antioxidant, protecting cells from damage caused by reactive oxygen species (ROS) under stress conditions [84]. ASA is a cofactor for various enzymes involved in antioxidant defence and other metabolic processes [85]. GR helps recycle oxidized GSH to its reduced form, replenishing the cell’s antioxidant defences under stress conditions [86]. These compounds play critical roles in maintaining cellular health and protecting against oxidative damage in plants under stress conditions. Biochemically, cobalt foliar treatment influenced protein, proline, glutathione (GSH), and ascorbate (ASA) contents in radish plants under salt stress conditions, suggesting improved stress tolerance and metabolic activity [87]. Cobalt may enhance protein synthesis, stimulate osmoprotectant accumulation, and modulate the antioxidant defence system, enhancing stress tolerance and overall plant health [88]. Its foliar application shows significant potential in alleviating the adverse effects of salinity stress on radish plants through multifaceted mechanisms. These mechanisms include growth promotion, photosynthesis enhancement, oxidative stress reduction, and modulation of stress-responsive biochemical pathways. These pathways involve regulating and adjusting various biochemical pathways within the plant in response to stress conditions. They encompass the alteration of gene expression, enzyme activity, and metabolite levels to assist the plant in adapting and coping with environmental stresses such as salinity [21, 89]. This pathway enables the plant to optimize its response mechanisms and maintain cellular homeostasis under challenging conditions. Through these mechanisms, cobalt supplementation proves effective in enhancing the salt stress tolerance of radish plants, as observed in various parameters studied. Our finding also validates the above arguments, where a significant increase in chlorophyll contents and growth attributes was noted where cobalt sulphate was applied as sol amendment in different levels with proline. The highest results are observed when applying 20 CoSO_4_ in radish plants, particularly in terms of proline content.

### Proline

Proline, a natural compound found in plants, plays a crucial role in helping them cope with stressful conditions like salinity [90, 91]. When plants face salinity stress, they accumulate proline as a protective mechanism. Proline acts as an osmolyte, helping to regulate water balance within the plant cells [92, 93]. It also serves as an antioxidant, scavenging harmful reactive oxygen species (ROS) that can damage cellular structures [94]. Salinity stress occurs when soil or water contains high salt levels, which can disrupt normal plant functions [95]. Under salinity stress, radish plants experience reduced growth, chlorophyll content, and cellular damage due to oxidative stress [74].

However, several positive effects are observed when proline is applied as a foliar spray to radish plants experiencing salinity stress. Proline helps to maintain shoot and root length, as well as plant fresh and dry weight. Salinity stress significantly reduces shoot and root length and the fresh and dry weight of radish plants. Proline foliar application mitigates these effects by promoting elongation and biomass accumulation [96]. Proline acts as an osmolyte, regulating water balance and maintaining turgor pressure, thus facilitating root and shoot growth even under stressful conditions [32]. It also preserves chlorophyll and carotenoid levels, which are essential for photosynthesis. Salinity stress decreases chlorophyll and carotenoid contents, impairing photosynthetic efficiency [97]. Proline foliar application helps maintain chlorophyll and carotenoid levels, possibly by stabilizing thylakoid membranes and preserving pigment synthesis pathways [92]. This ensures optimal light absorption and energy transfer, crucial for photosynthesis. Proline foliar application enhances the plant’s ability to retain water and reduces damage to cell membranes caused by electrolyte leakage under salinity stress [98, 99]. It also helps maintain protein levels within the cells and prevents oxidative damage to cellular proteins. Reduced water content (RWC) and increased electrolyte leakage (EL) are indicators of cellular dehydration and membrane damage under salinity stress. Proline application enhances RWC and reduces EL, suggesting improved water retention and membrane integrity [100]. Proline’s role as an osmoprotectant and antioxidant helps scavenge reactive oxygen species (ROS) and lipid peroxidation products, thus preserving cellular structure and function [101].

Furthermore, proline boosts the activity of antioxidant enzymes. These enzymes are crucial in scavenging ROS and maintaining cellular redox balance [94]. Antioxidant enzyme activities such as superoxide dismutase (SOD), peroxidase (POD), catalase (CAT), ascorbate peroxidase (APX), and glutathione reductase (GR) increase in response to salinity stress to counteract ROS accumulation. Proline application enhances antioxidant enzyme activities, facilitating ROS scavenging and redox regulation [102]. Proline’s role as an antioxidant and osmoprotectant supports antioxidant enzyme function, ensuring cellular redox homeostasis and stress tolerance. Salinity stress often leads to protein degradation and carbonylation, indicative of oxidative damage to cellular proteins of plant cells [103]. Proline application maintains protein content and reduces carbonyl levels, likely by stabilizing protein structures and inhibiting ROS-mediated protein modifications [104]. Proline ability to scavenge ROS and regulate redox balance contributes to protein homeostasis under stress. Salinity stress induces proline accumulation, which serves as a compatible solute and ROS scavenger to protect cellular structures [94]. Proline application further increases proline content, enhancing osmotic adjustment and ROS detoxification. Proline also influences the levels of other antioxidants such as reduced glutathione (GSH) and ascorbate (ASA), contributing to overall stress tolerance mechanisms. Proline foliar application effectively alleviates salinity stress in radish plants by promoting growth, maintaining photosynthetic efficiency, preserving cellular integrity, regulating protein homeostasis, enhancing antioxidant defenses, and modulating osmotic balance and redox status [105]. These mechanisms collectively contribute to improved stress tolerance and overall plant health in salinity-stressed environments.

In the current study, applying cobalt sulfate (20 CuSO_4_) combined with Proline foliar application proves to be an effective strategy for mitigating the harmful effects of salinity stress on radish plants. This combined treatment helps to preserve plant growth, photosynthetic capacity, and cellular integrity under challenging environmental conditions, such as salinity stress.

## Conclusion

The study concludes that applying cobalt sulfate with proline demonstrates the potential to reduce salinity stress in radish plants. Applying cobalt sulfate (20 mg/L) with proline as a foliar treatment appears more effective in enhancing vegetable growth and increasing nutrient concentrations in radish plants under salinity stress. Growers are recommended to utilize this foliar application of cobalt sulfate with proline to improve chlorophyll content and regulate antioxidants such as POD, SOD, CAT, and APX in radish plants facing salinity stress. Further investigations at the field level on various crops are necessary to confirm the efficacy of cobalt sulfate (20 mg/L) with proline (0.25 mM) as the optimal treatment for alleviating salinity stress.

## Data Availability

All data generated or analysed during this study are included in this published article.
